# Toll-like Receptors from the Perspective of Cancer Treatment

**DOI:** 10.3390/cancers12020297

**Published:** 2020-01-27

**Authors:** Nasir Javaid, Sangdun Choi

**Affiliations:** Department of Molecular Science and Technology, Ajou University, Suwon 16499, Korea; nasirjavaid1989@gmail.com

**Keywords:** toll-like receptor, pathogen-associated molecular pattern, cytokine, cancer, anticancer drug

## Abstract

Toll-like receptors (TLRs) represent a family of pattern recognition receptors that recognize certain pathogen-associated molecular patterns and damage-associated molecular patterns. TLRs are highly interesting to researchers including immunologists because of the involvement in various diseases including cancers, allergies, autoimmunity, infections, and inflammation. After ligand engagement, TLRs trigger multiple signaling pathways involving nuclear factor-κB (NF-κB), interferon-regulatory factors (IRFs), and mitogen-activated protein kinases (MAPKs) for the production of various cytokines that play an important role in diseases like cancer. TLR activation in immune as well as cancer cells may prevent the formation and growth of a tumor. Nonetheless, under certain conditions, either hyperactivation or hypoactivation of TLRs supports the survival and metastasis of a tumor. Therefore, the design of TLR-targeting agonists as well as antagonists is a promising immunotherapeutic approach to cancer. In this review, we mainly describe TLRs, their involvement in cancer, and their promising properties for anticancer drug discovery.

## 1. Introduction

Toll-like receptors (TLRs) belong to the family of pathogen recognition receptors (PRRs) in the innate immune system and recognize pathogen-associated molecular patterns (PAMPs) and damage-associated molecular patterns (DAMPs) related to foreign invading pathogens and host cells, respectively. To date, 13 TLRs have been reported in mice and 10 in humans, which are expressed on various immune cells (dendritic cells (DCs), macrophages, T-cell subsets, and B cells) and nonimmune cells (epithelial cells and fibroblasts) in humans. Based on their subcellular localization in humans, TLRs can be subdivided into two main groups: TLRs 1, 2, 4, 5, and 6 are present on the plasma membrane of cells; in contrast, TLRs 3, 7, 8, and 9 are localized in the endosomal membrane [1]. Each type of TLR recognizes its specific ligand(s) and activates the associated signaling pathway in either a myeloid differentiation primary response protein 88 (MyD88)- or TIR domain–containing adaptor inducing IFNβ (TRIF)-dependent manner. The activation of signaling leads to the secretion of various cytokines, which help the host body to combat various invaders. Furthermore, TLRs are responsible for maturation of DCs, which link innate and adaptive immune responses [2].

TLRs belong to the class of integral membrane type I glycoproteins, which have three major domains: the extracellular domain with different numbers of leucine-rich repeat (LRR) motifs, the transmembrane domain, and the cytoplasmic domain (similar to that of interleukin-1 receptor; IL-1R), which is known as the Toll/IL-1R (TIR) domain [3]. The extracellular domain has 19–25 tandem LRR motifs (20–26 in case of mammals), and each one of them contains 24–29 amino acid residues, including conserved residues (XØXXØXXXXFXXLX; Ø = hydrophobic residue) as well as motif XLXXLXLXX. Each LRR motif forms an α-helix and β-strand separated by a loop, which eventually forms a horseshoe structure after ligand binding on its inner concave surface in most of TLRs except TLR3, where the ligand binds to the outer convex surface [4].

Upon ligand recognition, TLRs undergo conformational changes to form homo- or heterodimers, which induce their TIR domain to interact with the TIR domain of intracellular adaptor molecules including MyD88, TIR domain–containing adaptor protein (TIRAP, i.e., Mal), TRIF, i.e., TICAM1, and TRIF-related adaptor molecule (TRAM, i.e., TICAM2) [5]. The details of this signaling pathway are described elsewhere [6]. Briefly, relevant adaptor molecules recruit various members of the interleukin 1 receptor–associated kinase (IRAK) family, in turn activating tumor necrosis factor receptor–associated factor 6 (TRAF6), whose ubiquitination switches on transforming growth factor β (TGF-β)-activated protein kinase 1 (TAK1). The latter causes the inhibitor of κB kinase (IKK) complex to stimulate the activity of a transcription factor (nuclear factor kappa-light-chain enhancer of activated B cells; NF-κB) and various mitogen-activated protein kinases (MAPKs) to trigger c-Jun N-terminal kinase (JNK), protein 38 (p38), and extracellular signal–regulated kinase (ERK), which turns on a transcription factor called activated protein 1 (AP-1) [7]. TLR3 and TLR4 employ TRIF/TRAM adaptor molecules to launch the activity of interferon response factor (IRF) 3, whereas TLR7, TLR8, and TLR9 activate IRF7. These transcription factors move into the nucleus and initiate the expression of various target genes including inflammatory cytokines, chemokines, and type I interferons (IFNs).

**Figure 1:**
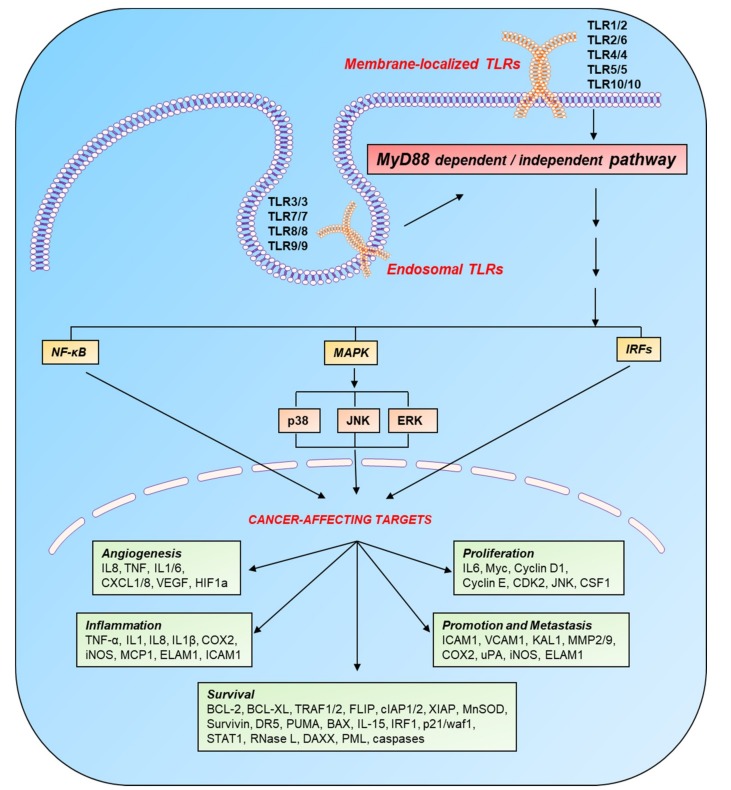
Induction of cancer-affecting genes by TLR signaling. TLRs are localized on the cell surface and in the endosomal compartment and become active after recognizing their respective PAMPS and DAMPs. On the basis of intracellular adaptor molecules, TLR pathways are categorized into two main cascades: MyD88-dependent and MyD88-independent. These pathways switch on various transcription factors: p50/p65, AP-1, and IRFs through NF-κB, MAPK, and IFN pathways, respectively. These transcription factors target various genes (involved in the processes of inflammation, angiogenesis, cell survival, proliferation, and metastasis), which directly or indirectly affect the progression of cancer. Legend: AP-1, activated protein 1; BAX, BCL2-associated X; BCL, B-cell lymphoma protein; CDK, cyclin-dependent kinase; cIAP, cellular inhibitor of apoptosis protein; COX, cyclooxygenase; CSF, colony-stimulating factor; CXCL, chemokine (C-X-C motif) ligand; DAXX, death domain–associated protein; DR, death receptor; ELAM, endothelial-leukocyte adhesion molecule; ERK, extracellular signal–regulated kinase; FLIP, FLICE-like inhibitory protein; HIF, hypoxia-inducible factor; ICAM, intercellular adhesion molecule; IFN, interferon; IL, interleukin; iNOS, inducible NO synthase; IRF, interferon response factor; JNK, c-Jun N-terminal kinase; KAL, Kallmann syndrome gene; MAPK, mitogen-activated protein kinase; MCP, monocyte chemoattractant protein; MMP, matrix metalloproteinase; MnSOD, manganese superoxide dismutase; MyD88, myeloid differentiation primary response 88; NF-κB, nuclear factor κB; p38, protein 38; PML, promyelocytic leukemia protein; PUMA, p53-upregulated modulator of apoptosis; STAT, signal transducer and activator of transcription; TLR, Toll-like receptor; TNF-α, tumor necrosis factor α; TRAF, tumor necrosis factor receptor (TNF-R)-associated factor; uPA, urokinase-type plasminogen activator; VCAM, vascular cell adhesion molecule; VEGF, vascular endothelial growth factor; WAF, wild-type activating fragment; XIAP, x-linked inhibitor of apoptosis protein.

Cancer cells divide uncontrollably unlike the ~200 types of somatic cells in our body, which can double 50–60 times before entering the state of senescence [8]. The cancer hallmarks include tumor metastasis and angiogenesis and tumor cell survival and proliferation [9]. The conversion to malignancy and cancer progression involve downregulation of tumor suppressor genes and upregulation of proto-oncogenes and associated signaling pathways [9,10]. Moreover, several cellular and molecular mechanisms help tumors escape the body’s own natural immune response [11,12]. The importance of immune regulation for cancer progression can be explained by the presence of increased amounts of immunosuppressive factors and cells and by scarcity of immune-system–activating signals in a tumor microenvironment. Under this scenario, it is worthwhile to activate immune cells through the receptors on their surfaces; one of these receptors is a TLR. Its activation is a double-edged sword, i.e., the exact pro- or antitumor effect depends upon the type of TLR, the cell type expressing it, and the downstream signaling cascade in such cells. For anticancer therapies, TLR agonists are being explored as vaccine adjuvants for the stimulation of immune cells and promotion of inflammation. TLR activity also upregulates the expression of such costimulatory molecules as CD40, CD80 (B7.1), and CD86 (B7.2) and cytokines like IL-12, which stimulates other immune cells like T lymphocytes [2,13]. In contrast, TLR expression and triggering on other cells including cancer cells can lead to tumor growth [14]. Active TLR signaling regulates the expression of various genes involved in tumor progression (Figure 1). Here, we review the roles of TLRs and their downstream pathways in cancer in relation to therapeutic interventions. 

## 2. The NF-κB Pathway 

The NF-κB family consists of five transcription factors—RelA (p65), RelB, c-Rel, p50 (fragment of p105) (NF-κB1), and p52 (fragment of p100) (NF-κB2)—which bind to the promoter region of their target genes in the form of homodimers or heterodimers. Stimulation of NF-κB is performed by various factors, e.g., cytokines (IL-1β, TNF-α), viral and bacterial products [dsRNA, lipopolysaccharide (LPS)], growth factors [epidermal growth factor (EGF)], reactive oxygen species (ROS), ionizing and UV radiation, and DNA damage stress from the affected cells. All these stimuli activate the inhibitor of κB (IκB) complex, which consists of three key components: NF-kappa-B essential modulator (NEMO) such as IKKγ, IKK1 (i.e., IKKα), and IKK2 (also known as IKKβ). Active IKK phosphorylates IκB at serines 32 and 36 for further processing.

The SCF-βTrCP E3 ligase (Skp1-Cul1-F-box ligase containing the F-box protein βTrCP) [15] and E2 of the Ubc4/5 family [16] take part in the ubiquitination of IκB. Two β-transducin repeat–containing proteins (β-TrCP), i.e., β-TrCP1 and β-TrCP2, have been discovered in mammalian cells. Their WD40 repeats at the C terminus interact with phosphorylated IκB, while the F-box interacts with the Skp, Cullin, F-box containing complex (SCF) complex containing Roc1/Rbx1 (RING domain protein), which interacts with E2 of Ubc4/5 allowing IκB ubiquitination on the two conserved lysine residues at its N terminus. This ubiquitinated IκB stays associated with NF-κB followed by selective degradation by the 26S proteasome, which eventually releases the NF-κB [17]. The released dimers relocate to the nucleus for induction of target genes.

NF-κB activation involves canonical (classic), non-canonical (non-classic), and alternative (atypical) pathways. The major pathway is the canonical one in most of cell types and involves p65, p50, and c-Rel. The pathway itself consists of IKK, IκB, and NF-κB, which is switched on by TLRs or other PRRs, proinflammatory cytokines (e.g., TNF-α and IL-1β), or cellular stressors [18]. It can also be triggered by DNA-damaging agents like etoposide, camptothecin, adriamycin, and ionizing radiation. IKKγ is phosphorylated by a sensor of DNA damage called ataxia telangiectasia mutated (ATM) and is recruited for the formation of the PIDDosome in the nucleus. Upon activation by ATM, NEMO moves to the cytoplasm and binds to IKKβ, which induces IκB degradation and launches the canonical pathway [19,20,21,22]. The non-canonical pathway includes TNF receptor family members such as B-cell–activating factor (BAF), lymphotoxin beta (LTβ), CD40, and viral proteins. This cascade involves NF-κB–inducing kinase (NIK)-dependent activation of IKKα with subsequent formation of p50 by the cleavage of p100. This p52 and RelB are translocated to the nucleus in the form of a functional complex and induce the expression of target genes [18,23]. Mediators of the atypical pathway (such as UV light of short wavelength) make NF-κB active in an IKK-independent manner by initiating IκB phosphorylation and degradation by casein kinase 2 (CK2) and calpain, respectively [24]. Similarly, hydrogen peroxide–mediated activation of NF-κB involves IκB phosphorylation at Tyr24 by the c-Src or Syk kinase [25,26].

NF-κB plays various roles by regulating transcription, apoptosis, and cell proliferation. It controls transcription mostly by acting as an inducer, with some exceptions. It induces ~200 genes involved in various functions like inflammation, immune responses, and growth [27]. Conversely, it suppresses the expression of certain genes when it is turned on by DNA-damaging drugs [28]. This suppressive behavior is cell line dependent and could occur after interaction with tumor suppressors or transcriptional repressors, e.g., ARF or p53 [29,30]. NF-κB regulates apoptosis by targeting some antiapoptotic proteins such as TRAF1, TRAF2, cIAP-1, cIAP-2, Bcl-xL, MnSOD, XIAP, and IEX-1L [31]. It can act as a proapoptotic factor by inducing the expression of mediators involved in apoptosis such as FAS ligand, death receptor DR5, Bax, and PUMA [32,33,34]. NF-κB regulates cell proliferation by transactivating the expression of c-Myc and cyclin D1. Of note, inflammation- and proliferation-triggering cytokines (such as TNF-α, IL-8, and IL-1β) are also expressed as a result of NF-κB activation [31]. Nonetheless, NF-κB can inhibit cell proliferation by suppressing JNK (a proliferation factor) and upregulating p21 (also known as WAF1; a cell cycle suppressor) [27]. 

Genes involved in cell survival and cell proliferation are known to be modulated by NF-κB [35]. Disruption of NF-κB–encoding and IκB-encoding genes owing to cancer-associated mutations, deletions, and chromosomal translocations might uncouple NF-κB from its regulators to make it constitutively active [36]. Constitutively activated NF-κB leads to cancer initiation via either cell proliferation or apoptosis inhibition through several mechanisms [37,38]. These involve transcriptional regulation of apoptosis-inhibiting proteins, e.g., XIAP, cIAPs, Bcl-2, and Bcl-xL [39,40]. NF-κB participates in protumor inflammation by means of certain cytokines, e.g., IL-1, TNF-α, and IL-6 as well as COX2, MCP1, and iNOS. Some of the target genes cause epithelial–mesenchymal transition (Twist, vimentin), angiogenesis-mediated remodeling of the extracellular matrix (IL-8, VEGF), and enhancement of metastasis and invasion (uPA, MMP2, and MMP9) [41]. Besides, NF-κB targets cyclin-coding genes such as cyclins G1, D1, and E [42,43].

## 3. The MAPK Pathway

The evolutionarily conserved MAPK pathway responds to extracellular signals and controls vital cellular processes including proliferation, growth, differentiation, apoptosis, and migration. Diverse stimuli trigger multiple MAPK pathways in a well-coordinated and integrated manner. These stimuli include growth factors, hormones, cytokines, TGF-β–associated agents, agents related to G protein–coupled receptors, environmental stress, DAMPs, and PAMPs. These pathways involve three kinase components: activation of MAPK results from its phosphorylation by MAPK kinase (MAPKK), and activation of MAPKK results from its phosphorylation by MAPKK kinase (MAPKKK). This phosphorylation affects Tyr and Thr residues of the Thr-X-Tyr motif in the activation loop of kinase subdomain VIII [44]. In mammals, six unique MAPK groups have been described: Jun N-terminal kinase (JNK) 1/2/3, p38 isoforms α/β/γ (ERK6)/δ, extracellular signal–regulated kinase (ERK) 1/2, ERK3/4, ERK5, and ERK7/8 [45,46]. Cells undergo tumorigenesis as a consequence of one or more processes: apoptosis evasion, independence from proliferative signals, continuous replication, non-responsiveness to antigrowth signals, invasion, metastasis, sustainability of angiogenesis, drug resistance, and evasion of oncogene-induced senescence [9]. These processes are triggered by abnormalities in MAPK signaling, which lead to cancer initiation and progression.

### 3.1. ERKs

Multiple MAPK pathways along with NF-κB are activated by inflammatory and stressful stimuli instead of mitogens. That is why they are promising drug targets in case of inflammation. Among all mammalian MAPKs, ERKs were the first to be identified. They are mostly considered mitogen- and insulin-activated MAPKs that are recruited by Ras-activating agonists. Ras next interacts with a protein of the Raf family, which switches on two MAP2Ks (MEK1 and MEK2). Detailed regulation and biology of the activation of Ras-dependent ERKs are reviewed elsewhere [47,48].

In many cases, the activation of ERKs is independent of Ras, but not when they are triggered by proinflammatory stimuli such as a cytokine (e.g., from the TNF family), a PAMP (e.g., lipopolysaccharides of invading pathogens), or a DAMP (e.g., oxidized low-density lipoprotein). PAMPs or DAMPs interact with PRRs (including TLRs) to turn on the ERK pathway that plays an important role in inflammation and innate immunity. Two genes, *Erk1* and *Erk2*, encode ERKs. These ERKs undergo dual phosphorylation at Tyr and Thr residues of subdomain VIII: Thr203-Glu-Tyr205 (ERK1) or Thr185-Glu-Tyr187 (ERK2) [48]. 

Several nuclear and cytoplasmic targets including phosphatases, kinases, cytoskeletal proteins, and transcription factors are phosphorylated by active ERKs [49]. Depending on the cell type, such processes as differentiation, chromatin remodeling, proliferation, migration, cell survival, and angiogenesis are regulated by ERK signaling [49,50]. Organized execution of these diverse processes by ERKs is based on differential expression and phosphorylation of their early targets like Jun, Fos, Egr-1, and Myc [51]. Continuous stimulation of ERK signaling promotes entry into the cell cycle via phosphorylation and stabilization of the above-mentioned targets as well as upregulation of genes needed for cell cycle entry (e.g., cyclin D1) and downregulation of antiproliferative genes [52]. Conversely, strong ERK signaling can induce the expression of p21 and p27 (CDK inhibitor proteins), which can cause cell cycle arrest [53]. Tumor cells counteract ERK-mediated stimulation of CDK inhibitor proteins by making AKT pathways constitutively active or by enhancing Rho signaling [53,54]. Additionally, ERK signaling promotes cell proliferation by counteracting the effect of TGF-β and other similar ligands. Nevertheless, various negative feedback loops maintain the balance between ERK activation and deactivation, whose deregulation may result in the initiation of cancer [55,56].

### 3.2. JNKs

JNKs were first reported as protein kinases in a cycloheximide-treated rat liver. These enzymes can phosphorylate Ser63 and Ser73 residues of transcription factor c-Jun; these residues are important for regulation of the transactivating function of AP-1 and c-Jun [57]. They are activated by mitogens, environmental stressors (oxidants, ionizing radiation, and heat shock), ischemia-reperfusion injury, genotoxins (alkylating agents and topoisomerase inhibitors), vasoactive peptides, mechanical shear stress, PAMPs, DAMPs, and proinflammatory cytokines [58,59,60]. Tunicamycin is an inhibitor of N-linked protein glycosylation and thereby stimulates JNKs. The reason is endoplasmic reticulum (ER) stress caused by the accumulation of misfolded proteins within the ER lumen. This observation indicates effective association of JNKs with ER stress, which can also be triggered by a high-fat diet leading to inflammation and insulin resistance. 

Three genes, *Jnk1–3* (also known as *Mapk8–10*, respectively), encode JNKs; among them, *Jnk1* and *Jnk2* are ubiquitous, whereas *Jnk3* is limited to the heart, brain, and testes [58,59,61]. The expression of these genes is subject to differential heterogeneous-nuclear-RNA splicing affecting (1) the catalytic domain extending to subdomains IX and X (to generate α and β JNKs, respectively) and (2) the terminal COOH region thus giving rise to 46 and 54 kDa polypeptides (named type 1 and type 2 JNKs), which might generate 12 JNK polypeptides. Each of these isoforms possesses the Thr-X-Tyr phosphoacceptor motif in its kinase subdomain VIII just as other MAPKs do; however, the sequence is Thr183-Pro-Tyr185 for JNKs. The α and β JNKs show slight variation in affinity for their substrates [58,60,62,63]. 

JNK activity and phosphorylated c-Jun play a major role in Ras-mediated tumorigenesis, and c-Jun and Ras cooperate for cellular transformation [64]. The phosphorylation site in c-Jun is the same for both Ras and JNK; therefore, Ras-mediated transformation is not observed in c-Jun–deficient fibroblasts [65]. It has also been reported that c-Jun can downregulate the *p53* gene [66]. Nonetheless, studies on JNK1/2-null cells have revealed that Ras-mediated transformation and tumorigenesis are independent of these two kinases. On the contrary, a JNK might promote apoptosis because of a tumor-suppressive function [67]. After exposure of cells to genotoxic drugs, JNK inhibitors impede DNA repair; hence, they can be employed for cancer therapy [68]. Nevertheless, their usefulness is not clear because of their ability to prevent apoptosis. The activation of NF-κB along with a JNK reverses the effect of the JNK because the former competitively inhibits the oncogene-induced apoptosis caused by the latter [69]. The reason is probably NF-κB–mediated induction of genes whose products repress JNK activity [70]. Therefore, apoptosis can be promoted in a JNK-dependent manner via inhibition of NF-κB activity. 

### 3.3. p38

The first isoform of p38 (p38α) was isolated using antiphosphotyrosine beads from the extracts of cells treated with endotoxin. Sequencing revealed its resemblance to osmosensing MAPK HOG1 from *Streptomyces cerevisiae* in terms of its phosphoacceptor motif Thr-Gly-Tyr [71,72]. Furthermore, it has been independently identified as an IL-1–activated and stress-activated kinase that can switch MK2 on after the phosphorylation of its particular element. MK2 (being a member of the Ser/Thr kinase family) in turn can make small heat shock protein Hsp27 active by phosphorylating it [73,74]. 

Isoforms of p38 are encoded by four genes: *MAPK14* (p38α), *MAPK11* (p38β), *MAPK12* (p38γ), and *MAPK13* (p38δ), which are effectively upregulated by inflammatory cytokines, environmental stress, PAMPs, and DAMPs [74,75,76,77,78]. The α isoform of p38 has been studied as an anti-inflammatory drug target of pyridinyl-imidazole compound SB203580 [78]. In vitro assays have revealed that SB203580 and its derivatives can inhibit only isoforms p38α and p38β because of the presence of the Thr106 residue in the pivot of the ATP-binding pocket in p38α and p38β [75,79,80,81].

In addition to the stress response, the p38 pathway participates in cell cycle progression and regulation of apoptosis, differentiation, and growth. A possible explanation is its responsiveness to diverse stimuli such as hormones and growth factors (like fibroblast growth factor, GM-CSFD, nerve growth factor, PDGF, and insulin-like growth factor 1). These stimuli lead to the activation of various MAPKKKs such as ASK1/2, MEKK4, TAK1, TAO1/2/3, MLK2/3, and DLK [82]. 

The tumor-suppressive function of p38 has been revealed in mice with disrupted p38α or both *Mek3* and *Mek6* [83,84]. The Ras-mediated transformation is also affected by the suppression of p38 activity [85]. p38 initiates p53-mediated apoptosis by acting as a negative regulator of cell cycle progression [86]. The oncogenic-stress–mediated induction of p38 in mouse embryonic fibroblasts has been reported to cause Ras-mediated senescence [87]. These findings point to the possibility of chemotherapeutic targeting of p38 for tumorigenesis suppression [88,89]. The inhibition of p38 enhances apoptosis after application of a DNA-damaging agent (such as cisplatin or doxorubicin) and a microtubule-disrupting agent (such as vinblastine, vincristine, or taxol) [90,91,92].

## 4. The Type I IFN Pathway 

The IFN family, originally discovered as a group of antiviral agents, is now known to start various biological processes in a cell-dependent manner. Their antiviral efficiency varies ~1000-fold, and some weak IFNs (like IFNγ) inhibit proliferation of specialized cells but not proliferation of other cell types, where they regulate migration, differentiation, activation, and apoptosis, e.g., in all immune effector cells. IFNs are categorized into type I IFNs (more than 20 subtypes including α, β, and ε), the single type II IFN (γ), and three type III IFNs (λ subtypes) according to their sequence, stimuli, receptors, and affected cell types. Proliferative stimulation of natural killer (NK) cells and NK T cells by cytokines (such as IL-12) yields IFNγ production. On the other hand, type I IFNs and IFNλs are produced as a result of viral infection. Despite differences, IFNs induce some common genes by triggering common pathways such as Janus kinase–signal transducers and activators of transcription (JAK-STAT). Among these IFNs, type I IFNs are exclusively induced by TLRs.

Type I IFNs belong to the class of the most diverse, evolutionarily conserved, and multigene cytokines of various subtypes. In humans, chromosome arm 9p contains a cluster of 30 types of IFN genes: 13 IFNα genes and single IFNβ, ω, κ, and ε subtypes with 13 pseudogenes [93]. The *IFNε* gene has a specific expression pattern being silent in hematopoietic-origin tissues [93]. In humans and mice, IFNκ is basically expressed by keratinocytes along with DCs and monocytes.

Most of IFNαs share 78–98% identity, with 166 amino acid residues folded into five α-helices with two disulfide bonds for stabilization. IFNβ and IFNε show almost 35% identity to the IFNα amino acid sequence. Human IFNα is only weakly glycosylated, in contrast to both IFNβ and murine IFNαs [94]. Most of type I IFNs are highly stable at 56 °C and pH 2 with isoelectric points of 5.6 and 8.9 for IFNα and IFNβ, respectively [95]. To understand their pharmacokinetics, it is important to know their physicochemical properties. IFNα has somewhat higher stability (2–3 h half-life) in serum than IFNβ does; the latter is rarely detectable because of its hydrophobic nature even though it induces a response of the magnitude comparable to that of IFNα. These properties suggest that IFNα may be more effective systemically, whereas IFNβ may be more effective locally by functioning in a paracrine or autocrine manner.

Type I IFNs perform a variety of functions: (1) They allow cells to combat a viral infection whose mechanism is cell- and virus-dependent. IFN-regulatory genes target various stages of viral replication such as entry, transcription, translation, maturation, assembly, and the final release. For example, viral RNA is degraded by host RNase L, which is activated by the 2′-to-5′-linked oligoadenylates produced by 2′–5′ oligoadenylate synthetase in an IFN-dependent manner [96]. Similarly, translation and replication of a virus are blocked in an IFN-dependent manner by protein kinase R (PKR) and Mx proteins, respectively [97,98]. (2) IFNs play an antiproliferative role by affecting cell cycle regulators such as c-Myc [99] and the production of growth factors and their associated receptors [100]. The proliferation of bone marrow–derived macrophages is inhibited by IFNβ with the help of colony-stimulating factor 1 (CSF1) [101]. Type I IFNs inhibit myelopoiesis because greater numbers of circulating myeloid cells are observed in IFNAR1-null mice [102]. The influence on proliferation is actually cell type dependent because IFNs stimulate the survival and/or proliferation of memory T cells, the effect that might be mediated by IL-15 [103]. (3) Type I IFNs affect the expression of apoptosis/survival-regulating molecules such as IRF1, p21, OKR, STAT1, RNase L, DAXX, PML, caspases, TNF family members, BCL2 family members, and death-associated kinases [104,105,106,107,108,109]. (4) Type I IFNs participate in immunoregulation by affecting the cells of innate and adaptive immune systems. These IFNs enhance the survival and differentiation of T cells [110], immature myeloid DCs [111], and B cells [112] as well as in vitro and in vivo cytotoxicity, cell proliferation, and cell differentiation in an NK cell–mediated manner [113]. Additionally, IFNs control immune cells’ recruitment at an inflammatory site by inducing chemokines and associated receptors such as CCL12 and CXCL10 [114,115].

## 5. TLR Signaling in Immune and Cancer Cells 

**Figure 2:**
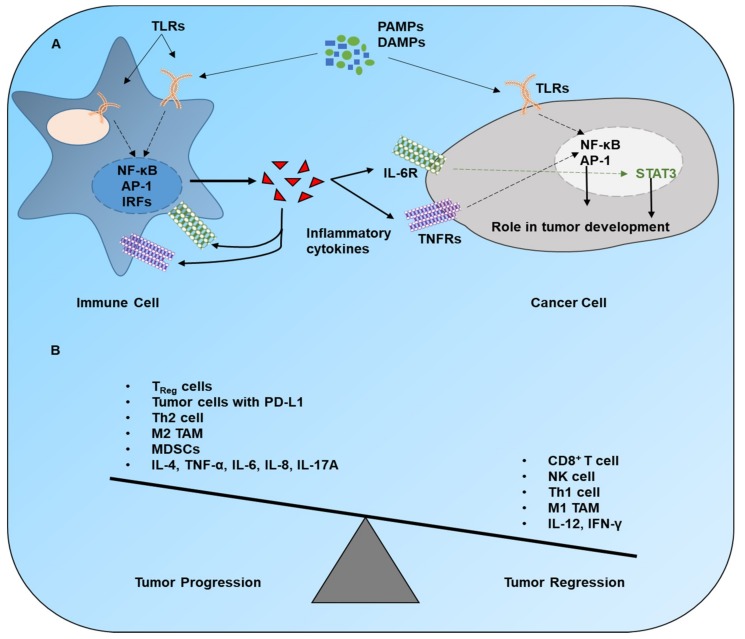
Immune regulation of cancer progression. (**A**) The stimulation of TLRs and other PRRs in immune cells launches downstream signaling pathways, which cause a release of various cytokines. These cytokines interact with their receptors on immune cells and cancer cells to trigger associated signaling pathways. The product(s) of these cascades plays a substantial role in the progression of cancer. (**B**) The overall outcome of cancer in a tumor microenvironment depends upon the ratio of protumor to antitumor signals. Legend: CD, cluster of differentiation; DAMPs, damage-associated molecular patterns; IFN, interferon; IL, interleukin; MDSCs, myeloid-derived suppressor cells; NK, natural killer; PAMPs, pathogen-associated molecular patterns; PD-L1, programmed-death ligand 1; TAM, tumor-associated macrophage; Th2, T helper type 2; TNF-α, tumor necrosis factor α; Treg, T regulatory.

The pro- or antitumor effect of TLR signaling is determined by the specific TLR being stimulated, the cell type with activated signaling, and a downstream signaling cascade in the activated cells. Various TLR agonists promote inflammation by activating immune cells and are currently tested in clinical trials of anticancer therapies as vaccine adjuvants. TLRs are expressed on many cell types in humans but are mainly detectable on DCs, monocytes, and mature macrophages [116]. Ligand engagement by TLRs on these cells causes overexpression of multiple membrane-bound costimulatory molecules like CD40, B7.1 (CD80), and B7.2 (CD86) along with cytokines needed for proper T-cell activation such as IL-12 [2,13]. Aside from TLR activation on antigen-presenting cells (APCs), TLR activation in other cells also plays a substantial part in tumor growth. For instance, tumor cells and T cells express various TLRs that are activated after recognition of their associated ligands [14,117]. Proper communication between immune and cancer cells via cytokines or costimulatory molecules exerts an antitumor effect (Figure 2).

Treatment of mouse models with TLR agonists lessens the growth of tumors and even destroys established tumors in some cases during a combinatorial therapy with other agents such as monoclonal antibodies, chemotherapy drugs, and antigenic vaccines, e.g., plasmid DNA, peptides, or proteins [118,119,120,121,122,123]. 

The expression profile of TLRs on T cells depends upon the T-cell subset in question as well as their activation status. Lower levels of TLR mRNA and protein are detected in naïve T cells and increase dramatically upon their stimulation via TCR or by such compounds as PMA or ionomycin [124]. Similarly, stand-alone activation of TLRs has a smaller effect on resting or naïve T cells, but TLRs exert costimulatory effects on T cells during the stimulation of TCR [125,126]. Nonetheless, TLR expression is transient and diminishes with the passage of time [124,125,127]. Notably, some TLRs are expressed more weakly in human and murine memory T cells than in activated T cells, but this level is enough for them to respond in the absence of triggering by TCR [128,129].

### 5.1. TLR Signaling in DC Subsets

In the immune system, DCs are considered most efficient professional APCs. Immature DCs undergo infection- or inflammation-mediated activation and differentiation into mature DCs that activate the cells of adaptive immunity such as B and T lymphocytes [130]. The maturation of DCs involves a series of steps such as reduced changes in the sets of receptors of endocytosis and phagocytosis; overexpression of costimulatory molecules including CD40, CD58, and CD86; morphological changes; and reshuffling of lysosomal and MHC compartments. The DC population is a heterogeneous collection of various subtypes that differ in their function, phenotype, and localization. Two main populations are found in peripheral human blood, which include CD11c-negative plasmacytoid DCs (pDCs), CD11c-positive myeloid DCs (mDCs), monocyte-derived DCs (moDCs), and CD34^+^ cell–derived DCs [131]. These subsets express distinctive PRRs, which allow them to perform specialized functions by detecting different pathogenic stimuli. DCs take up pathogens and present pathogen-derived processed peptides to T cells by means of major histocompatibility complex (MHC) molecules. The activation status of DCs determines the overall outcome of an immune response. For example, resting DCs or those receiving inhibitory signals (such as corticosteroids or IL-10) promote immune tolerance by inducing upregulation of regulatory T (T_reg_) cells or elimination of effector T cells; however, mature DCs evoke immunity. T cells are activated after recognition of the peptides processed and expressed by DCs (signal 1) and stimulation by cytokines (signal 2) and costimulatory molecules (signal 3).

TLR expression on DCs depends upon subtypes, species, and maturation stage. All three subpopulations of mDCs (i.e., CD1c^−^ mDCs, CD16^−^ mDCs, and BDCA3^−^ mDCs) express TLRs 1–10 except TLR3 (absent only in CD16^−^ mDCs) at the RNA level. Both CD1c^−^ mDCs and CD16^−^ mDCs respond strongly to agonists of all TLRs except agonists of TLR9 (CpG oligodeoxynucleotides) [132]. Of note, Cd16^−^ mDCs respond to the TLR3 ligand poly(I:C) despite the absence of *TLR3* RNA, in a TLR3-independent manner, possibly because of either cytosolic RNA sensors [133] or minor contamination with endotoxins. The CD1c^−^ mDCs and moDCs both express similar endosomal (TLR3 and TLR8) and extracellular TLRs (TLRs 1, 2, 4, 5, and 6), which allow them to produce inflammatory cytokines after stimulation by the respective ligands [134,135]. Nonetheless, both subsets do not express TLR9, whereas TLR10 is expressed only by mDCs [135,136]. On the other hand, pDCs express TLR1 weakly, which is nonresponsive to the ligand of TLR1/2 owing to the absence of TLR2 [135]. Despite the absence of TLR3 and TLR8 expression, pDCs respond to viral pathogens, probably via TLR7, which binds to the same ligand and shares the signaling pathway. In addition, pDCs express TLR10 whose binding partner and function are still unknown [137,138].

The distinct profile of TLRs in various subsets indicates that mDCs mainly respond to fungal and bacterial antigens, whereas pDCs mainly respond to viral pathogens. These subsets can be used in DC vaccination therapy for an antitumor and Th1 response [136,139,140]. Human pDCs can infiltrate various tumors such as ovarian cancer [141], head and neck cancer [142], and breast cancer [143]. The differentiation and maturation of infiltrating pDCs are prevented by the suppressive environment created by soluble factors secreted from a tumor [144,145,146]. Despite infiltration, pDCs cannot become activated after sensing DNA at the site of infiltration. These phenomena lead to the induction of T_reg_ cells and a poor prognosis [143,147]. Other studies have revealed that the recruitment of pDCs and the production of type I IFNs can be enhanced by a TLR7 agonist (imiquimod), which causes tumor regression by creating an inflammatory environment [148,149]. Similarly, an antitumor response has been observed in melanoma skin metastases and basal cell carcinoma after pDC activation by intratumoral injection of a TLR9 agonist, a CpG oligodeoxynucleotide [150]. This agonist will activate only pDCs because of the absence of TLR9 on mDCs. By contrast, a TLR7/8 ligand (R848) can stimulate both pDCs and mDCs, and this approach will be more effective in eliciting an antitumor response at the tumor site.

Recent studies showed a cooperative and synergistic association between mDCs and pDCs. Along with direct induction of a CD8^+^ T-cell response specific to a tumor antigen, pDCs stimulate the tumor antigen-presenting ability of mDCs toward T cells [151]. Moreover, both of these human DC subsets stimulate each other when any of them is activated in vitro by its respective TLR ligand [152]. In clinical settings, the research in this field suggests that DC vaccination with both mDCs and pDCs is more effective than vaccination with moDCs alone in terms of an antitumor response [152].

### 5.2. TLR Signaling in T-Cell Subsets

#### 5.2.1. TLR1/2 and TLR2/6

The functional engagement of TLR1/2 on CD8^+^ cells enhances the production of TNF-α [153,154], IFNγ [155], and IL-2 [153] along with cytolytic molecules like perforin and granzyme B [156]. The importance of T-cell–mediated TLR signaling is emphasized by reversed or delayed tumor growth of B16 melanoma in tumor-bearing MyD88 knockout mice that received adoptive transfer of TCR-transgenic CD8^+^ pmel T cells, after peritumoral injections of Pam3CSK4 (a synthetic TLR2 agonist) [157]. In vitro and in vivo models have confirmed that a bacterial lipoprotein can kill tumor cells too by recruiting CD8^+^ cells [158,159].

Furthermore, the T-cell response is directly modulated by some DAMPs such as heat shock proteins. For example, CD45RA^+^ naive and CD45RO^+^ memory T cells show reduced chemotaxis and increased β1-integrin–dependent adhesion after downregulation of two chemokine receptors, CCR7 and CXCR4, because of Hsp60-mediated stimulation of TLR2 present on the surface of these cells [160]. The interactions between T cells and tumor cells or APCs are mediated by the presence of integrins on T cells, and these integrins strengthen the cell activation [161] and are considered the markers distinguishing between effector and memory T-cell subsets [162].

A durable and effective immune response is hindered by the tolerance and immune suppression caused by T_reg_ cells. The cytolytic activity of tumor-specific CD8^+^ T cells is compromised by the production of TGF-β and IL-10. Moreover, the suppressive function of T_reg_ cells is reduced by the stimulation of their TLR2 because CD8^+^ T cells proliferate when grown in coculture with T_reg_ cells treated with a bacterial lipoprotein [155]. Likewise, in in vitro and in vivo studies on murine T_reg_ cells, stimulated-TLR2–mediated reversal of the suppression is reported to be mediated by IL-2 and TCR activation [163,164,165,166].

Low-affinity tumor antigens cause insufficient stimulation of TCR signaling thereby posing another problem for the effective antitumor response of T cells [167,168]. Some other studies indicate formation of memory cells owing to weak TCR signaling after TLR1/2 activation on CD8^+^ cells [128,157,169]. Increased PKC and PI3K signaling activities cause costimulation of TLR signaling [170,171]. An increase in the expression of T-bet and its enhanced binding to the promoter regions of granzyme B, IFNγ, and perforin genes is observed in CD8^+^ cells after TLR stimulation [156]. The importance of TCR activation is proven by the experiments showing that melanoma tumor growth is reduced by the injection of TCR-transgenic CD8^+^ pmel T cells and of the TLR2 ligand Pam3CSK4 into tumor-bearing MyD88 knockout mice; this phenomenon is not observed in the mice injected only with pmel T cells and MyD88 knockout pmel or TLR2 knockout pmel T cells [157]. Other studies have highlighted possible usefulness of lipopeptides as vaccines to generate a broad-spectrum T-cell repertoire via the triggering of TLR signaling in T cells [172]. Enhanced degranulation and IFNγ production have been observed in γδT cells after TLR activation through TCR [173].

Increased levels of IFNγ and IL-2 are observed in CD4^+^ and CD45RO^+^ cells after TLR2 stimulation [126,153]. Memory CD4^+^ T cells can be directly stimulated by TLR2-agonistic lipoproteins of *Mycobacterium tuberculosis* that cause enhanced proliferation and production of IFNγ and IL-2. Along with TLR2 stimulation, these cells require associated TCR signaling for the proper response [174]. Moreover, TLR2 agonists increase the longevity of T cells through downregulation of proapoptotic proteins like Bim and upregulation of antiapoptotic proteins such as Bcl-xL and A1 [126,157]. 

#### 5.2.2. TLR3

TLR3 is expressed by activated CD4^+^ T cells and enhances NF-κB–dependent cell survival and proliferation upon stimulation with a specific ligand, poly(I:C). The enhanced survival of cells is attributed to increased expression of antiapoptotic protein Bcl-xL [175]. It is reported that poly(I:C) can enhance the response and proliferation of CD8^+^ T cells independently of CD4^+^ T cells and APCs [158]. Moreover, the formation of memory T cells is enhanced by TLR3 costimulation via TCR; this phenomenon is attributed to the prolonged T-cell survival due to TLR3 signaling. In T cells, the ability of TLR3 signaling to obviate CD4- or APC-mediated costimulation and formation of memory T cells is a helpful feature for the design of cancer vaccines because costimulatory signals are absent in a tumor microenvironment [129].

Among CD8^+^ T cells of humans, IFNγ production by PHA-activated memory or effector T cells rises after stimulation with poly(I:C) but fails to increase their lytic activity [176]. Similarly, proliferation of mouse CD8^+^ T cells and their secretion of IFNγ were augmented after preincubation with antigen-pulsed splenocytes with poly(I:C). Unlike untreated CD8^+^ T cells, those treated with the TLR3 ligand showed a greater expansion potential upon adoptive transfer and displayed higher amounts of high-affinity CD25 (IL-2R α-chain) and activation marker CD69 [177]. The enhanced expression of CD69 and increased production of IFNγ have been observed in TLR3-stimulated freshly isolated γδT cells [178]. In vitro, granzyme A–mediated and granzyme B–mediated cytolytic activities of an expanding γδT cell population are augmented after TCR stimulation by bromohydrin pyrophosphate and pretreatment with poly(I:C) [179]. Along with IFN-γ, TLR3 activation in T-cells might also lead to the secretion of IFN-β which plays a main role in antiviral response [180,181,182].

#### 5.2.3. TLR4

The activation and greater proliferation of T cells under the influence of LPS are mediated by stimulation of the production of a cascade of proinflammatory cytokines by APCs [183]. LPS-induced TLR4 stimulation is also observed in CD4^+^ and CD8^+^ T cells that can produce TNF-α, IFNγ, granzyme B, and perforin [184,185]. On the contrary, the expression of TLR4 and CD14 has not been detected in murine CD8^+^ T cells [185]. Nonetheless, murine naïve CD4^+^ T cells show increased survival and proliferation upon LPS treatment unlike nonresponsive naïve murine T cells [186]. Thorough analysis has revealed that *TLR4* mRNA is expressed by the Th17 subset of murine CD4^+^ T cells and that LPS stimulation increases the level of IL-17A and reduces that of IFNγ because of decreased activation of MAPK [187,188]. The activation of these cells by TLR4 can lead to colitis by aggravating intestinal inflammation.

LPS-induced activation of TLR4 in CD4^+^CD25^+^ T_reg_ cells enhances immunosuppressive activity and proliferation unlike the engagement of TLR1/2 [189]. On the other hand, HMGB1-induced triggering of TLR4 on T_reg_ cells reduces IL-10 production and the expression of forkhead box p3 and CTLA4 [171]. Furthermore, switched-on TLR4 in T_reg_ cells mainly launches signaling in a TRIF-dependent manner, but in nonregulatory T cells, this process is mediated by MyD88 and p38 MAPK [189]. These studies highlight the ligand- and cell-dependent response after TLR4 engagement.

#### 5.2.4. TLR5

TLR5 binding by its ligand (bacterial flagellin)—similarly to other TLR agonists—leads to the production of IL-8, IL-10, and IFNγ but not IL-4. The costimulatory effect of flagellin on effector memory CD4^+^CCR7^−^ cells is stronger than the effect on CCR7^+^ central memory cells [190]. The induction of IFNγ in the absence of IL-4 elicits a Th1 reaction that facilitates an efficient response of CD8^+^ T cells. The increased proliferation and production of TNF-α, IFNγ, and granzyme B under the influence of flagellin have also been observed in CD8^+^ T cells of human cord blood. This response is surprisingly stronger when it is implemented in combination with Pam3CSK4 (a TLR2 agonist) [190]. These synergistic effects of various TLR ligands highlight their usefulness in a combinatorial therapy designed to evoke an in vivo antitumor response of T cells. The activation of TLR5 on human CD4^+^CD25^+^ T_reg_ cells enhances the expansion of this subset with augmented suppressive activity in contrast to the inhibitory effects of other TLR agonists on murine T_reg_ cells [191]. Nevertheless, it is necessary to confirm these results in vivo and elucidate the production of T_reg_ cell–inhibiting cytokines by macrophages and DCs.

#### 5.2.5. TLR7/8

Human T_reg_ cells express TLR8, but naïve CD4^+^ T cells do not. A TLR9 ligand, CpG-A, induces the production of IFNα and IFNβ, which facilitate the proliferation of effector CD4^+^ T cells by reversing the suppressive actions of T_reg_ cells. Furthermore, an enhanced antitumor activity with a loss of suppressive activity by T_reg_ cells is observed in a tumor mouse model upon their adoptive transfer after pretreatment with poly-G10 (a TLR8 ligand) [192]. Additionally, this study indicates that the TLR9 expressed on T_reg_ cells can recognize CpG DNA molecules too [192]. Similarly, TLR8 activation on human suppressor γδT cells by ssRNA40 or poly-G3 reverses their in vivo and in vitro suppressive effects on CD4^+^ T cells [193]. The activation of TLR7/8 in human CD4^+^ T helper cells by its synthetic ligand such as resiquimod (R-848) increases the production of IL-10, IL-2, and IFNγ with enhanced proliferation independent from APCs [190].

#### 5.2.6. TLR9

The survival and antitumor response of CD4^+^ T cells are increased by TLR9 stimulation [164]. The increased in vitro survival of TLR9-activated murine T cells is explained by the initiation of NF-κB signaling and enhanced expression of antiapoptotic protein Bcl-XL [175]. It is reported that costimulation of T cells by a TLR9 ligand enables them to overcome their reliance on PKC-ϕ signaling and reverses their anergy status by re-establishing in vitro survival and proliferation [194]. Moreover, the activation of CD4^+^ T cells by a TLR9 ligand makes them resistant to the suppressive effect of T_reg_ cells [195]. Additionally, TLR9 engagement increases the number of CD4^+^ and CD8^+^ T cells by boosting the expression of IL-2 and of its associated receptor IL-12R, which takes place even in the absence of costimulatory molecules (such as CD28) in a tumor microenvironment [196]. A reduction in radiation-induced apoptosis and increased DNA repair are observed in TLR9-activated CD4^+^ T cells [197].

### 5.3. TLR Signaling in the Cancer Cell

TLRs are mainly expressed by immune cells such as macrophages, DCs, and T-cell subsets. Recent studies uncovered the expression of TLRs in various tumor cells [198,199,200,201,202]. For instance, the majority of colon cancer cells overexpress TLR2, TLR3, and TLR4 [203,204]. Similarly, ovarian cancer cells overexpress TLR2, TLR3, TLR4, and TLR5 [205,206].

Researchers are focusing on the expression and function of TLRs in various cancers. Enhanced invasiveness of human gastric cells and greater vascularization of gastric tissue after the activation of TLR2 enhance tumor growth by inducing the production of IL-8, PGE2, and COX-2 [207]. Higher mRNA copy numbers of *TLR3* are observed in the colon mucosa of polyposis patients; this parameter is linked to the stages of colorectal cancer [208]. A reduction in the incidence, size, and number of neoplasms induced by chemicals has been observed in TLR4- and MyD88-deficient mice, thus underscoring a supportive role of TLR signaling in hepatocarcinogenesis [159,209]. The progression of human breast cancer is strengthened by the production of immunosuppressive factors (e.g., NO, IL-6, IL-12, VEGF, and MMPs) after the engagement of TLR4 by its ligand [210,211,212]. In mouse models of colon cancer, stimulation of TLR4 leads to the overexpression of an ICOS ligand (B7-H2) and programmed cell death ligand 1 (B7-H1) as well as downregulation of death receptor Fas; these changes prolong tumor survival [213]. The activation of TLR5 in human gastric cancers causes production of IL-8 and TNF-α, which lead to the proliferation of tumor cells [214]. The expression of TLR5 and TLR9 is significantly increased in late-stage cervical cancer but not observed in normal cervical squamous epithelial cells [215]. TLR7/8 activity enhances tumor growth, survival, metastasis, and inflammation in lung cancer patients [216]. TLR9 expression enhances angiogenesis, which is linked to lower survival rates of lung cancer patients [217]. Moreover, time- and dose-dependent proliferation of prostate cancer cells is observed during TLR9-mediated expression of NF-κB and c-Myc [218].

Although the incidence and progression of various tumors are promoted by TLRs, some TLRs may possess an antitumor function. *Mycobacterium* Bacillus Calmette-Guérin (BCG) is enriched with peptidoglycans and unmethylated CG-containing DNA, which stimulates TLR2, TLR4, and TLR9. Treatment with BCG reduces motility and proliferation and increases the apoptosis of cells of urothelial carcinomas [219]. Inhibition of proliferation and promotion of apoptosis of prostate cancer cells are observed after the activation of protein kinases by an agonist of TLR3, poly(I:C) [220]. Apoptosis of human colon cancer cells has been observed after combinatorial treatment with poly(I:C) and either IFNα or 5-fluorouracil [221]. Increased TLR3 expression in human melanoma cells after pretreatment with a type I IFN results in the inhibition of proliferation with subsequent death of these tumor cells [222]. The evidence of antitumor activity of TLR4 is scarce; however, its triggering on lung epithelial cells has a protective effect against the formation of a lung tumor [223]. 

TLR5 signaling in breast cancer downregulates cyclins B1, D1, and E2 thus inhibiting the proliferation of the tumor cells [224]. Increased apoptosis and decreased proliferation of head and neck cancer cells are seen after treatment with a TLR5 agonist, flagellin [225]. Cell cycle arrest and reduced proliferation of human glioma cells are reported after the launch of downstream NO and NF-κB pathways by a TLR9 agonist (CpG ODN 107) and irradiation [226]. Moreover, TLR9 activity enhances apoptosis of neuroblastoma cells and inhibits the angiogenesis in renal cell carcinoma [227].

In a word, cells of various cancer types express various TLRs. Among them, TLR3 and TLR5 have more promising antitumor effects unlike TLR4, -7, -8, and -9. Of note, activation of a particular TLR on one type of tumor cells has an antitumor impact but may play a protumor role in another tumor type(s). Therefore, it is important to choose an optimal TLR agonist for tumor cells in question in order to ensure an antitumor effect. This choice should be based on the expression profile of TLRs and their functional outcome for this type of cancer. 

## 6. TLRs as Therapeutic Targets in Cancers

The therapeutic targeting of TLRs in cancer has become more complicated with the discovery of protumorigenic activities of several TLRs in certain cancer types and the resultant clinical setbacks [228]. The activation of similar kinds of TLRs may have a protumor (Table 1) or antitumor (Table 2) effect in different types of cancers. To determine the effect on a tumor exactly, it is necessary to confirm suitable tumor types for immunotherapy, suitable TLRs to be targeted, and combinatorial options to enhance the immune response. Most of the relevant clinical trials have evaluated the agonists of TLR3, TLR7/8, and TLR9. Similarly, antagonists of TLR2 and TLR4 or their cognate ligands have shown antitumor properties. 

### 6.1. TLR Agonism for Cancer Prevention or Treatment 

#### 6.1.1. TLR2/TLR4

Despite their protumor activity, TLR2 and TLR4 have been studied as components of adjuvants for vaccination and tumor therapy. For example, BCG switches on TLR2 and TLR4 thereby exerting antitumor immunomodulatory effects [263,264,265,266,267] especially in bladder cancer (FDA approval has already been obtained) [268,269,270]. Similarly, a derivative of *Escherichia coli* lipid A, OM-174 (CXR-526), engaging both TLR2 and TLR4, is being tested in a phase I trial against a solid tumor and phase I/II trials as a vaccine adjuvant for melanoma treatment. Stimuvax contains monophosphoryl lipid A, which stimulates TLR4; it is useful against the MUC1 tumor antigen but does not alleviate non–small cell lung carcinoma [271]. 

#### 6.1.2. TLR3

The TLR3 ligand poly(I:C) has shown antitumor effects in several mouse studies [272,273], but hardly any data are available regarding humans. Several alternative ligands of TLR3 are being developed because of the rapid degradation of poly(I:C). For example, a poly(I:C) derivative (poly-ICLC; Hiltonol®) is stabilized by poly-lysine and is being evaluated in a phase II clinical trial against a solid tumor. Another derivative, rintatolimod (Ampligen^®^), features a substitution of cytidine with uridine at a 1:12 ratio and is used in the treatment of fallopian tube, ovarian, and brain tumors in combination with some vaccines. The administration of TLR3 ligand (poly(I:C)) reduces orthotopic prostate cancer in transgenic mice (TRAMP C57B16 × FvB F1 Tg^+/−^) as well as TRAMP tumors subcutaneously implanted in syngenic mice [272]. Similarly, single administration of poly(I:C) into B16-F10-induced mouse model of metastatic lung cancer arrested tumor growth in association with greater influx of dendritic cells (DCs) which created cytotoxic immune environment [273].

#### 6.1.3. TLR5

A TLR5 agonist, flagellin, and TLR5-agonistic nanoparticles have shown significant antitumor effects in mice [224,244,274]. Entolimod (CBLB502) derived from *Salmonella* flagellin [275] is a TLR5 agonist that is in phase I clinical trials against squamous cell head and neck cancer and solid tumors. The treatment of breast cancer cells with TLR5 agonist (flagellin) activated the intrinsic signaling pathway which led to the inhibition of anchorage-independent growth and cell-proliferation [224]. Another study showed the contrasting result of flagellin administration into mice subcutaneously transplanted with weak immunogenic tumor or its variant stably expressing strong antigenic HER-2 oncoprotein. Administration of flagellin after 8-10 days of tumor implantation significantly reduced the growth of antigenic variant tumor but not that of weakly immunogenic. In contrast, flagellin administration with antigenic-tumor implantation accelerated its growth. These contrasting results are because of increased ratio of IFN-γ:IL-4 and decreased number of CD4^+^CD25^+^ T regulatory cells in first case and vice versa. The early combinatorial treatment of flagellin with CpG-containing oligodeoxynucleotides completely suppressed the tumor growth [244].

#### 6.1.4. TLR7/8

TLR7/8 are the most effective among all TLRs with respect to immunomodulatory anticancer effects. Hence, the only TLR agonist approved for cancer therapy is the one targeting TLR7/8, imiquimod. Both of these TLRs are activated simultaneously by the same ligand because of their common ability to recognize single-stranded RNA. The agonists of TLR7/8 have been classified into guanosine and adenosine analogs such as imiquimod and loxoribine, respectively. The ligands can be targeted specifically to TLR7 or TLR8 after modification of their RNA sequence [276]. Among all such ligands, imiquimod is in clinical use: the FDA and European Medicines Agency have approved Aldara (5% imiquimod cream) for the treatment of basal cell carcinoma, and this substance has a 42–100% clearance rate [277,278]. It has also found applications in the treatment of other local cutaneous tumors, including lentigo maligna, with a >85% success rate and significant clearance of melanoma [279]. Unlike imiquimod, 852A (a TLR7 agonist) and VTX-2337 (a TLR8 agonist) can be administered systemically and are being tested in phase I/II clinical trials against various malignant tumors, e.g., ovarian, breast, cervical, endometrial, and head and neck cancers.

#### 6.1.5. TLR9

Various TLR9 agonists based on CpG oligodeoxynucleotides are being tested in animal models of neuroblastoma, cervical carcinoma, and colon cancer [261,280,281,282,283] and in some clinical trials [284]. Despite good preclinical results, several trials have been disappointing: there were safety issues of IMO-2055 with platinum-based therapies in a phase II trial against recurrent and metastatic head and neck cancer, and a phase III trial of CPG7909 failed against non–small cell lung cancer [228]. As a combinatorial therapy, CPG7909 (a TLR9 agonist), monophosphoryl lipid A (a TLR4 agonist), and MAGE-A3 (a melanoma antigen) are currently evaluated in phase III clinical studies. 

### 6.2. TLR Antagonism for Cancer Treatment or Prevention 

The protumor effects of TLRs in such organs as the liver, colon, and pancreas necessitate inhibition of TLR signaling at these sites for cancer treatment. Unfortunately, the results of studies on animal models cannot be translated into clinical trials so far. Promising antagonistic strategies are discussed below. 

#### 6.2.1. Manipulation of the Gut Microbiota 

The microbiota of intestines is rich in bacterial TLR ligands, which substantially participate in the carcinogenesis of the colon, stomach, and liver. TLR-mediated tumor-promoting and inflammatory signals can be reduced by modulating the bacterial translocation and/or gut microbiota by means of antibiotics or probiotics [285]. In murine models of azoxymethane (AOM)-induced colon cancer, the formation of aberrant cryptic foci is prevented by synbiotics but not by a pro- or prebiotic alone [286,287]. Administration of probiotic VSL#3 in a rat model of liver carcinogenesis reduces the formation of a liver tumor [288]. In a genetic murine model of colorectal cancer, the expression of protumorigenic IL-23 in tumor-associated macrophages is reduced by short-term treatment with an antibiotic, whereas the number and size of tumors decrease with long-term suppression of the gut microbiota [289]. A reduction in the formation of colonic dysplasia is observed after sterilization of the gut in a colon cancer model [290]. The tumor burden in rat and mouse models is drastically reduced by sterilization of the gut with oral antibiotics [291,292]. The antibiotics are more effective if administered at later stages of hepatocarcinogenesis, indicating a possible role of the gut microbiota in cancer prevention, where early treatment is not possible. In murine hepatocarcinogenesis, a nonabsorbable well-tolerated antibiotic like rifaximin can decrease liver tumors [291] and is approved for the treatment of hepatic encephalopathy [293].

#### 6.2.2. Inhibition of TLR2 and TLR4 

Synthetic analogs derived from the lipid A portion of LPS (E5564 and CRX-526) inhibit TLR4 by preventing LPS binding to the TLR4–MD2 complex. The intracellular domain of TLR4 is targeted by another TLR4 inhibitor, TAK-242. Despite their inhibitory action on LPS-induced inflammation, they have not been tested for cancer prevention in either clinical trials or animal models [294,295,296]. Similarly, OPN305, a humanized monoclonal antibody, reduces in vivo inflammation but has not been tested regarding cancer prevention [297].

## 7. Conclusions

The prime purpose of TLRs is to help the human body to develop immunity by activating various downstream signaling pathways that result in the secretion of diverse proinflammatory cytokines. TLRs also perform a major function in tumor immunity by activating various cells such as DCs, T-cell subsets, and even tumor cells. This finding has led to the design of diverse TLR agonists as therapeutics against various cancers. Successful studies have involved a TLR7 agonist, imiquimod, and a nonspecific agonist of TLR2/TLR4, BCG. The discovery of new molecules, combinatorial therapies, and indications is in progress. An attractive possible tumor therapy is incorporation of TLR-specific agonists into cancer vaccination based on DCs [298]. Nonetheless, the activation of TLRs can also lead to inflammation that culminates in tumor promotion. Under this scenario, inhibition of TLR signaling may be useful for tumor regression. It is necessary to further study the “Yin–Yang” mechanisms of action of TLRs in tumor biology.

There is need to develop appropriate TLR-targeting drugs for the prevention/elimination of cancer. Several types of drug molecules exist based on their biochemical nature such as protein, small molecules, and aptamers. Each of them has its own benefits and drawbacks which affect physicochemical and pharmacokinetic properties of the associated drug. Discovering an appropriate drug is quite lengthy and complex process which involves target selection and its validation; compound screening and lead optimization; preclinical studies; and clinical trials. Target selection and library screening involve various computational approaches such as analysis of genome and proteome; high-throughput screening; virtual screening; and combinatorial chemistry. These traditional approaches are time-consuming and expensive. It would be better choice to use state-of-the-art and most advanced approaches like artificial intelligence (AI) for the screening of TLR-targeting compounds. More efficient and specific drug will lead to better and safe prevention of TLR-associated cancers with fewer side-effects.

## Figures and Tables

**Table 1:** TLRs with a protumor effect.

TLRs	Agonist/Ligand	Mechanism	Cancer Type	Enhanced Cancer Characteristics	References
TLR2	Peptidoglycan (PGN)	Synergistic effect of wound-associated injury and PGN	Epithelial ovarian cancer	Self-renewal, repair, and recurrence	[229]
	Versican	Inflammatory microenvironment	Lewis lung carcinoma	Metastasis	[230]
		hCAP18/LL-37 overexpression	Ovarian tumor	Growth and invasion	[231]
	pg-LPS	Increased NF-κB signaling; IL-6, TGF-β, VEGF, and MMP9 secretion	MDA-MB-231 breast cancer cells	Invasion	[232]
	Arg753Gln and (GT)n microsatellite polymorphisms	TLR2 overexpression and increased NF-κB signaling	Colorectal cancer	Growth, progression, and invasion	[233]
	−196 to −174del	Decreased transcription of *TLR2* gene	Breast cancer, gastric cancer, hepatocellular carcinoma	Tumor progression due to weaker immune response	[234,235,236]
	Bacterial PGN	Augmentation of NF-κB, STAT3, and Smad3 activities	Breast cancer	Invasion and adhesion	[237]
TLR4	LPS	Increased secretion of TGF-β, VEGF, and IL-8	Lung cancer, ovarian cancer	Immune evasion and apoptosis resistance	[238,239]
		Activation of PI3K–AKT signaling and promotion of β1 integrin function	Colorectal cancer	Increased adhesiveness and metastasis	[240]
		Increased mitochondrial ROS production	Gastric cancer, non–small cell lung cancer	Increased cell proliferation	[241,242]
		Increased NF-κB signaling	Pancreatic cancer	Increased invasion and progression	[243]
TLR5	Flagellin	Decreased IFNγ:IL-4 ratio and increased number of CD4^+^CD25^+^ T_reg_ cells	Tumor mouse model	Tumor growth	[244]
		Enhanced activity of NF-κB, IL-8, and ERK	Gastric cancer	Cell proliferation	[214]
TLR7/8	ssRNA	Activated NF-κB, upregulation of Bcl-2	Lung cancer	Survival and chemoresistance	[216]
	Loxoribine	Enhanced signaling	NSCLC	Progression and chemoresistance	[245]
	Resiquimod (R848)	Elevated NF-κB and COX2 expression	Pancreatic cancer	Proliferation and chemoresistance	[246]
TLR9	CpG ODN	Elevated expression of IL-1α, IL-8, CXCR4, ICAM1, and MMP2	Human lung cancer	Metastasis	[247,248]
		Greater response of NF-κB/RELA and STAT3 pathways	Prostate cancer	Cell proliferation	[249,250]

Abbreviations: BCL, B-cell lymphoma protein; CD, cluster of differentiation; COX, cyclooxygenase; CXCR, chemokine (C-X-C motif) receptor; ERK, extracellular signal–regulated kinase; hCAP, human cationic antimicrobial protein; ICAM, intercellular adhesion molecule; IFN, interferon; IL, interleukin; MMP, matrix metalloproteinase; NF-κB, nuclear factor κB; PI3K, phosphoinositide 3-kinase; RELA, REL-associated protein; ROS, reactive oxygen species; STAT, signal transducer and activator of transcription; TGF, transforming growth factor; TLR, Toll-like receptor; VEGF, vascular endothelial growth factor.

**Table 2:** TLRs with an antitumor effect.

TLRs	Agonist/Ligand	Mechanism	Cancer Type	Inhibited Cancer Characteristics	References
TLR2	MicroRNA-154	TLR2 downregulation at post-transcription level	Colorectal cancer	Tumor growth, migration, and invasion	[251]
	Krestin	Stimulation of CD8^+^ T cells and NK cells	Breast cancer	Growth	[154]
TLR3	Synthetic dsRNA	Elevated signaling	Breast cancer	Tumor survival	[252]
	Poly(I:C)	PI3K/AKT pathway and autophagy	Prostate cancer	Growth and survival	[253]
TLR4	DAMPs	Antitumor T cells response with activation of DCs	Colorectal cancer	Cell proliferation	[254]
	Angelan	Enhanced DC maturation	Melanoma	Tumor growth	[255]
TLR5	Flagellin	Increased IFNγ:IL-4 ratio and decreased number of CD4^+^CD25^+^ T_reg_ cells	Tumor mouse model	Tumor growth	[244]
		CD8^+^ CTL immune responses	Tumor model	Growth and survival	[256]
		Increased MAP1S expression	Breast cancer	Tumor cell growth and migration	[257]
		Increased signaling	NSCLC	Cell proliferation, migration, and invasion	[258]
		Activated signaling	Breast cancer	Cell growth and proliferation	[224]
TLR7/8	Imiquimod	Establishment of proimmunogenic microenvironment	Breast cancer	Metastasis	[216]
	Resiquimod (R848)	Maturation and differentiation of MDSCs	Tumor model	Growth	[259]
	Imiquimod	Inhibition of nitric oxide synthase	Tumor model	Growth	[260]
TLR9	CpG ODN	Enhanced signaling	Neuroblastoma	Growth and survival	[261]
	PF-3512676	Enhanced signaling	Melanoma	Metastasis	[150,262]

Abbreviations: CTL, cytotoxic T lymphocyte; DCs, dendritic cells; dsRNA, double-stranded RNA; IFN, interferon; IL, interleukin; MAP1S, microtubule associated protein 1S; MDSCs, myeloid-derived suppressor cells; NK, natural killer; NSCLC: non–small cell lung cancer; ODN, oligodeoxynucleotide; PI3K, phosphoinositide 3-kinase; poly(I:C), polyinosinic:polycytidylic acid; T_reg_ cells, regulatory T cells.
